# Pleural effusion biomarkers and computed tomography findings in diagnosing malignant pleural mesothelioma: A retrospective study in a single center

**DOI:** 10.1371/journal.pone.0185850

**Published:** 2017-10-02

**Authors:** Takehiro Otoshi, Yuki Kataoka, Shunkichi Ikegaki, Emiko Saito, Hirotaka Matsumoto, Sawako Kaku, Masatoshi Shimada, Masataka Hirabayashi

**Affiliations:** Department of Respiratory Medicine, Hyogo Prefectural Amagasaki General Medical Center, Amagasaki, Hyogo, Japan; Baylor College of Medicine, UNITED STATES

## Abstract

In this study, we aimed to examine the clinical value of the pleural effusion (PE) biomarkers, soluble mesothelin-related peptide (SMRP), cytokeratin 19 fragment (CYFRA 21–1) and carcinoembryonic antigen (CEA), and the utility of combining chest computed tomography (CT) findings with these biomarkers, in diagnosing malignant pleural mesothelioma (MPM). We conducted a retrospective cohort study in a single center. Consecutive patients with undiagnosed pleural effusions who underwent PE analysis between September 2014 and August 2016 were reviewed. This study included 240 patients (32 with MPM and 208 non-MPM). SMRP and the CYFRA 21-1/CEA ratio had a sensitivity and specificity for diagnosing MPM of 56.3% and 86.5%, and 87.5% and 74.0%, respectively. Using receiver operating characteristics (ROC) curve analysis of the ability of these markers to distinguish MPM from all other PE causes, the area under the ROC curve (AUC) for SMRP and the CYFRA 21-1/CEA ratio was 0.804 and 0.874, respectively. The sensitivity and specificity of SMRP combined with the CYFRA 21-1/CEA ratio were 93.8% and 64.9%, respectively. The sensitivity of the combination of SMRP, the CYFRA 21-1/CEA ratio, and the presence of Leung’s criteria (a chest CT finding that is suggestive of malignant pleural disease) was 93.8%. In conclusion, the combined PE biomarkers had a high sensitivity for diagnosing MPM, although the addition of chest CT findings did not improve the sensitivity of SMRP combined with the CYFRA 21-1/CEA ratio. Combination of these biomarkers helped to rule out MPM effectively among patients at high risk of suffering MPM and would be valuable especially for old frail patients who have difficulty in undergoing invasive procedures such as thoracoscopy.

## Introduction

Malignant pleural mesothelioma (MPM) is a highly aggressive tumor, and it is estimated that its incidence will reach a peak between 2015 and 2025 in many countries [1]. The association between MPM and asbestos exposure has been studied [2], and a previous report from Japan showed that the standardized mortality ratio of people living within 1500 m from a now-closed asbestos cement pipe plant was 4.3 [3].

It is often difficult to diagnose MPM because of the low sensitivity of pleural effusion (PE) cytology [4]. This difficulty in MPM diagnosis is the reason why invasive diagnostic procedures such as pleural biopsy with video-assisted thoracoscopy are often required for the definite diagnosis of MPM.

Currently, more than half of MPM patients are 70 years or older [5]. For some old frail patients, it is difficult to undergo invasive procedures such as thoracoscopy. Among those old frail patients at high risk of suffering MPM, simple methods to rule out MPM with high accuracy would be valuable for their follow-up. Cytokeratin 19 fragment (CYFRA 21–1) and carcinoembryonic antigen (CEA) have been applied for the differential diagnosis of MPM, and it has been reported that elevated CYFRA 21–1 levels with low CEA levels in PE are highly suggestive of MPM, or that MPM should be suspected when the ratio of CYFRA 21–1 to CEA in PE (CYFRA 21-1/CEA ratio) is elevated [6,7]. Furthermore, the utility of assessment of the PE levels of soluble mesothelin-related peptide (SMRP), which is also known as mesothelin, in MPM diagnosis has been reported [1,4,8–20]. However, only two previous reports have assessed the diagnostic accuracy of simultaneous assessment of SMRP, CYFRA 21–1 and CEA when diagnosing MPM [9,18]. Moreover, no previous reports have analyzed if the addition of chest CT findings to these PE biomarkers might enhance the sensitivity in diagnosing MPM, even though chest CT findings are important when malignant pleural diseases are suspected [21].

In the present study, we investigated the diagnostic utility of the PE biomarkers, SMRP and the CYFRA 21-1/CEA ratio among patients with undiagnosed pleural effusions. Subsequently, we examined the sensitivity of chest CT findings combined with these PE biomarkers for diagnosing MPM.

## Materials and methods

### Study design

A retrospective study was performed in our hospital (700-bed teaching hospital in Amagasaki city, Japan). In July 2015, our hospital was moved to a new location. Both the current and the previous hospitals are located within 2000 m from the closed asbestos cement pipe plant in Amagasaki city. This retrospective study was in accordance with the Standards for the Reporting of Diagnostic Accuracy Studies (STARD) statement 2015, and was approved by the institutional review board of Hyogo Prefectural Amagasaki General Medical Center (approval number 28–63). All procedures performed in this study involving human participants were in accordance with the ethical standards of the institutional and national research committee and with the 1964 Helsinki declaration and its later amendments or comparable ethical standards. The institutional review board of our hospital waived the need for informed consent from patients involved in this study because of its retrospective design. Patients records were accessed anonymously.

### Subjects

**Fig 1:**
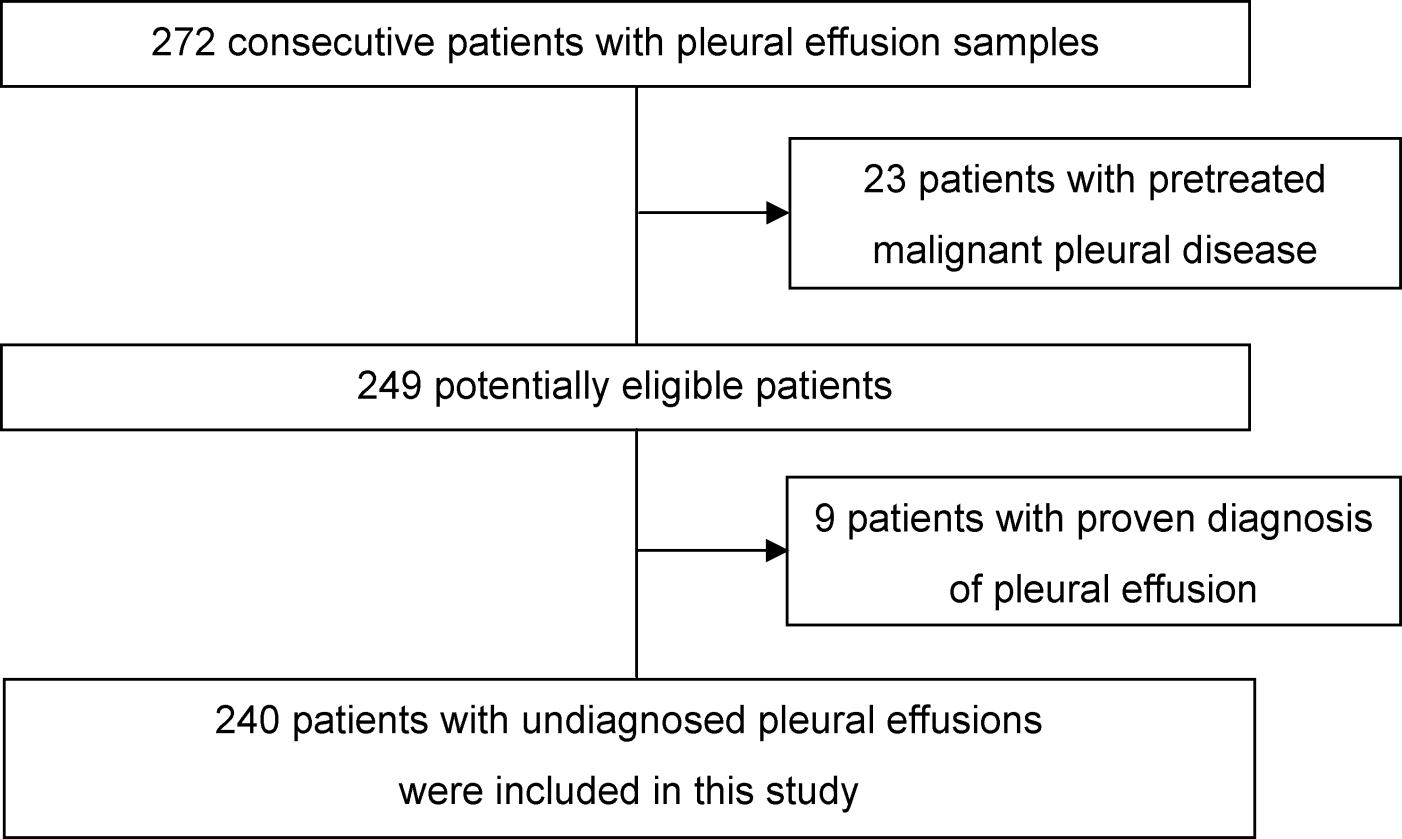
Flow chart of patient selection.

The medical records of consecutive inpatients or outpatients who underwent PE analysis at the Department of Respiratory Medicine between September 2014 and August 2016 were reviewed. Patients who had pretreated malignant pleural disease at the time of PE analysis were excluded from this study. Patients who had proven PE diagnosis at the time of PE analysis were also excluded, and patients with undiagnosed pleural effusions were included in this study (Fig 1).

### Assessment of chest CT findings

Chest CT findings were reviewed independently using Leung’s criteria [21] by two pulmonologists. According to Leung’s criteria, any of the following four features of pleural thickening is suggestive of malignant pleural disease: 1) circumferential pleural thickening; 2) nodular pleural thickening; 3) parietal pleural thickening greater than 1 cm; and 4) mediastinal pleural involvement [21]. Other reports have also suggested that these four features of pleural thickening are useful in the differential diagnosis of pleural diseases. For example, it was previously reported that these four features are the most common CT findings in MPM patients [22]. Another study reported that these four features are independent findings for the differential diagnosis of malignant pleural diseases from benign pleural diseases [23]. Each of these four features of pleural thickening was assessed (present or absent) by the same two pulmonologists blind to the PE diagnosis, and disagreements were resolved by discussion.

### Measurement of pleural effusion biomarkers

All specimens were received as fresh PE. PE samples were collected in standard blood collection tubes. Samples were centrifuged at 4000 rpm for 5 min, following which aliquots of the supernatants were used for analyses. SMRP concentrations were measured using the chemiluminescent enzyme immunoassay (CLEIA), Lumipulse Mesothelin^®^ (Fujirebio Inc., Tokyo, Japan), which is based on a 2-step sandwich method, following the manufacturer’s instructions. The detail of this method is well described in a previous report that used the same method as ours [14]. This method uses the same two anti-SMRP antibodies as those used in the commonly used SMRP enzyme-linked immunosorbent assay (ELISA), the MESOMARK^®^ kit (Fujirebio Diagnostics Inc., PA, USA). Nakamachi et al. reported that SMRP concentrations measured with Lumipulse Mesothelin^®^ were almost the same as those measured with MESOMARK^®^ in blood samples of 887 patients, and that the regression line was given by y = 1.07x-0.17, r = 0.999 (x, SMRP concentration measured with MESOMARK^®^; y, SMRP concentration measured with Lumipulse Mesothelin^®^; r, Pearson’s correlation coefficient) [24]. Therefore, the SMRP results of the present study can be directly compared with those of studies that assayed SMRP using MESOMARK^®^. The SMRP results are expressed in nanomoles per liter (nmol/L). CYFRA 21–1 was measured using the chemiluminescent immunoassay (CLIA), CYFRA Abbott^®^, (Abbott Japan Inc., Chiba, Japan). CEA was also measured with a CLIA, CEA Abbott^®^, (Abbott Japan Inc., Chiba, Japan). In brief, 50μl of sample (CYFRA 21–1) or 10μl of sample (CEA) were incubated with 50μl of microparticles at 37°C for 18 min. After washing, 50μl of conjugate were added and incubated at 37°C for 4min. After washing, 100μl of pre-trigger and 300μl of trigger were added, and chemiluminescent signal were measured. CYFRA 21–1 and CEA results are expressed in nanograms per milliliter (ng/mL). These biomarkers were determined blind of the PE diagnosis in a single laboratory analysis.

### Diagnosis of PE

The medical records of each patient were reviewed for the PE diagnosis. To confirm the diagnosis, the clinical data, clinical course and CT findings of patients included in this study were reviewed independently by two pulmonologists who were blind to the PE biomarkers. The final diagnosis was established by their consensus. Diagnostic criteria in this study were defined based on a previous report [1], and are described in Table 1. To confirm the histological diagnosis of MPM, immunostaining was performed. Strong reactivity for calretinin and cytokeratin 5/6 with negative reactivity for carcinoembryonic antigen (CEA), thyroid transcription factor-1 (TTF-1) and epithelial membrane antigen (EMA) was considered to be supportive of the diagnosis of MPM [17]. Light’s criteria were used to confirm transudates and exudates [25]. Tuberculosis pleuritis was diagnosed using adenosine deaminase (ADA) levels and the lymphocyte neutrophil ratio in PE, as described in a previous report [26].

**Table 1:** Diagnostic criteria.

Diagnosis of pleural effusion	Diagnostic criteria
Malignant pleural mesothelioma	Histological diagnosis from pleural tissue biopsy or cytological diagnosis from pleural effusion.
Lung cancer	Meet at least one of the following criteria. (1) Histological diagnosis from pleural tissue biopsy or cytological diagnosis from pleural effusion. (2) Histological or cytological diagnosis from extra-pleural specimen and radiographic evidence of pleural metastases.
Other malignancy	Meet at least one of the following criteria. (1) Histological diagnosis from pleural tissue biopsy or cytological diagnosis from pleural effusion. (2) Histological or cytological diagnosis from extra-pleural specimen and radiographic evidence of pleural metastases.
Unconfirmed malignant pleural effusion	Radiographic evidence of pleural malignancy in the absence of histological or cytological diagnosis.
Benign asbestos exposure-related pleural effusion	Meet all the following criteria. (1) History of exposure to asbestos or radiographic evidence of pleural plaques. (2) Stable or improving chest CT findings during the follow-up period. (3) Benign histology of pleural tissue biopsy.
Cardiac cause	Meet all the following criteria. (1) Transudative effusion according to Light’s criteria. (2) Clinical evidence of cardiac failure (history of cardiac failure or evidence of left ventricular failure or moderate-severe valve disease on echocardiogram or improvement in effusion with diuretic therapy). (3) Absence of any other cause of pleural effusion.
Non-cardiac transudate	Meet all the following criteria. (1) Transudative effusion according to Light’s criteria. (2) Biochemical evidence of hepatic or renal failure or hypoalbuminemia. (3) Absence of any other cause of pleural effusion.
TB pleuritis	Meet any of the following criteria. (1) Culture positive from sputum, pleural effusion or pleural tissue biopsy, or classical pleural tissue histology. (2) ADA of 50 U/L or greater and lymphocyte neutrophil ratio of 0.75 or greater in pleural effusion.
Simple parapneumonic effusion	Meet all the following criteria. (1) Clinical presentation suggestive of respiratory infection. (2) Exudative effusion according to Light’s criteria. (3) Pleural effusion that is gram stain and culture negative with a PH>7.2. (4) Absence of loculation on chest CT.
Pleural infection	Meet all the following criteria. (1) Clinical presentation suggestive of respiratory infection. (2) Exudative effusion according to Light’s criteria. (3) A. Pleural effusion that is gram stain or culture positive or B. Pleural effusion with a PH≤7.2 or C. Pleural effusion with frank pleural pus or D. Presence of loculation on chest CT or E. Pleural infection confirmed by pleural biopsy histology or microbiological culture. (4) Absence of any other cause of pleural effusion.
Idiopathic pleuritis	Pleural tissue biopsy negative for malignancy and chest CT findings inconsistent with pleural malignancy.
Undiagnosed	None of the above criteria were reached within 12 month after pleural fluid examination.

TB, tuberculosis; ADA, adenosine deaminase.

### Data presentation and statistical analysis

Analyses were carried out using the statistical software, JMP 9.0.2 (SAS Institute Inc., Cary, NC, USA) and Stata® ver. 13.1 (Stata Corp., College Station, TX). Comparisons were performed using the t-test for continuous variables and the Chi-square test for categorical variables. We calculated weighted kappa statistics to evaluate the interobserver agreement in the quantitative assessment [27]. We used multiple imputation to handle missing data because we thought the missing values were occurred by missing at random. Twenty datasets were imputed by normal regression and estimates from these datasets were combined using Rubin’s rule. We used the imputed set for investigating the utility of chest CT findings and PE biomarkers in diagnosing MPM, while the complete-case set was also used for sensitivity analysis.

Differences in the distribution of PE biomarkers were compared between MPM patients and non-MPM patients. The sensitivity and specificity of PE biomarkers were evaluated by receiver operating characteristics (ROC) analysis, using the area under a ROC curve (AUC). The 95% confidence intervals (95% CI) of the AUC were estimated by bootstrap method, and we performed 500 iterations for the bootstrapping. We defined the cut-off level of SMRP a priori as 20 nmol/L based on previous reports [1,4,10,11]. For the assessment of CYFRA 21–1 and CEA, we used the CYFRA 21-1/CEA ratio, and defined the cut-off level of the CYFRA 21-1/CEA ratio a priori as 19.1 according to a previous report [7]. In all cases, p values ≤0.05 were considered significant.

## Results

### Patient characteristics

During the study period, a total of 272 consecutive patients with PE underwent PE examinations at the Department of Respiratory Medicine in our hospital. Of these patients, 23 patients were excluded as they had pretreated malignant pleural disease, and 9 patients were excluded as they had proven PE diagnosis. The diagnoses of the 32 patients excluded from this study were: MPM (n = 7); lung cancer (n = 22); other malignancy (n = 2); and others (n = 1). Two hundred and forty patients with undiagnosed pleural effusions were included in this study (Fig 1). Agreement between the two pulmonologists with regards to the PE diagnosis of patients included in this study was good with a weighted kappa concordance score of 0.982 (95%CI 0.946 to 1.000).

Of the 240 patients included in this study, 32 were MPM and 208 were non-MPM. Of the 32 MPM patients, 7 patients were diagnosed only by PE cytology, and 25 patients were diagnosed by pleural tissue histology (12 epithelioid, 1 sarcomatoid, 10 biphasic, and 2 other histological subtypes). Among the 7 MPM patients diagnosed only by PE cytology, 6 were diagnosed by cell block methods for immunohistochemical analysis. Among the 18 patients diagnosed as TB pleuritis according to the diagnostic criteria, 11 patients were diagnosed as TB pleuritis besed on the ADA and lymphocyte neutrophil ratio in pleural effusion. However, 3 of the 11 patients had no resolution of pleural effusion with anti TB therapy, and they were eventually defined as undiagnosed PE.

**Fig 2:**
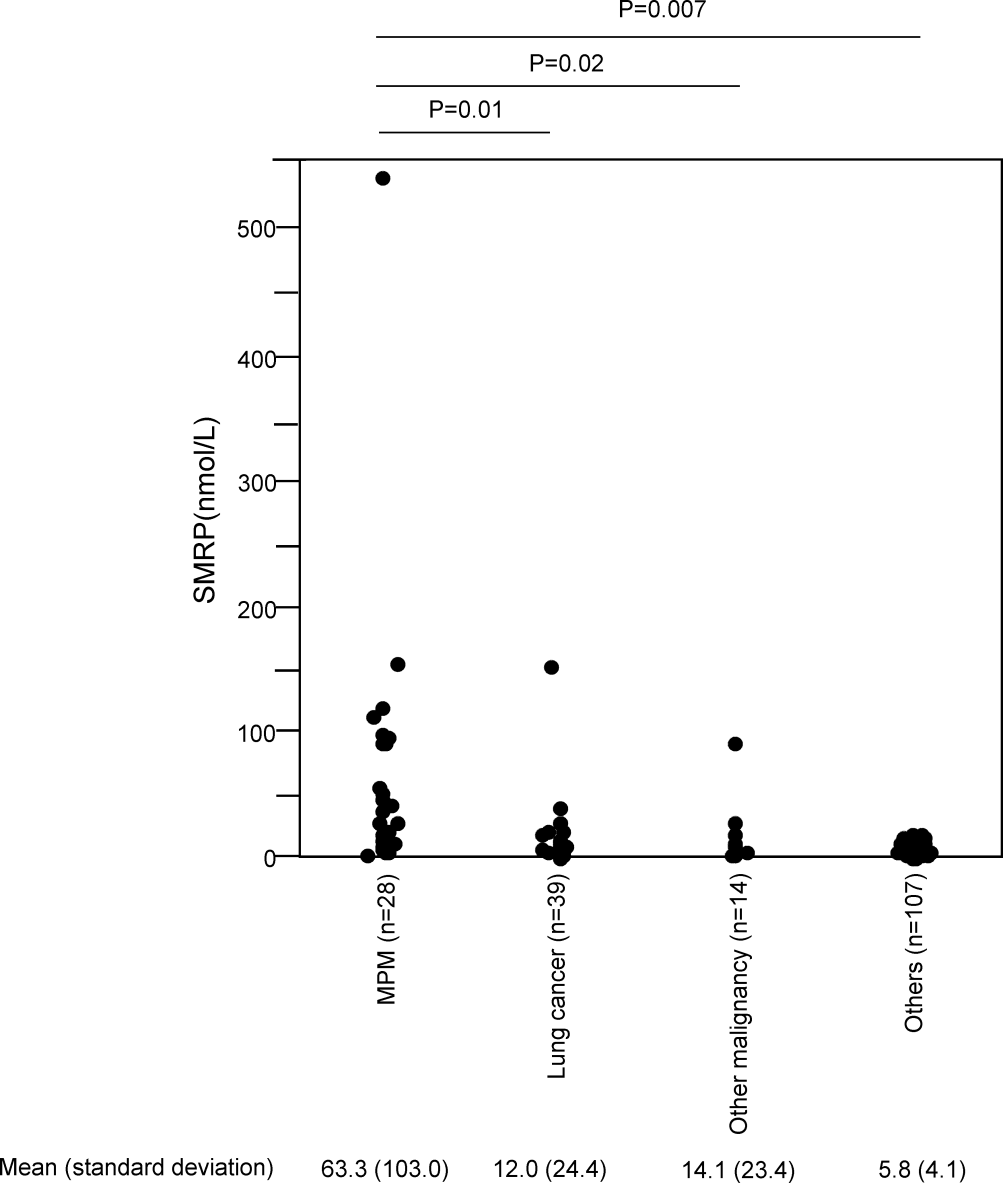
SMRP levels in each subgroup of patients.

**Fig 3:**
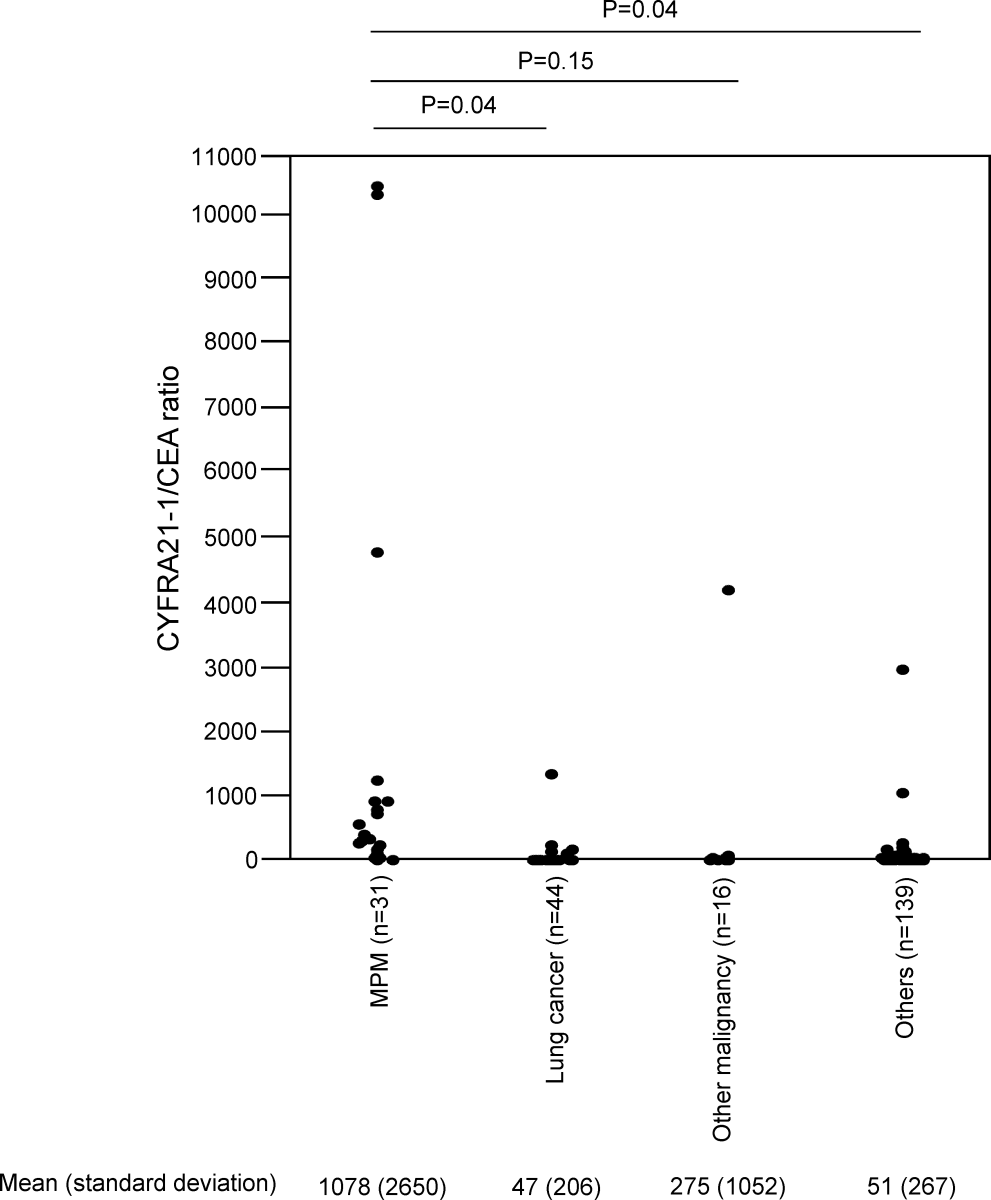
CYFRA 21-1/CEA ratio in each subgroup of patients.

SMRP levels and the CYFRA 21-1/CEA ratio in the 32 MPM patients and the 208 non-MPM patients are summarized in Table 2. The mean (standard deviation) SMRP level of the MPM patients was 56.1 (97.8) nmol/L, which was significantly greater than that of the non-MPM patients (8.7 (25.2) nmol/L; p<0.0001). The mean (standard deviation) CYFRA 21-1/CEA ratio of the MPM patients was 1044 (2572), which was significantly greater than that of the non-MPM patients (63.1 (394) nmol/L; p<0.0001). The mean (standard deviation) SMRP levels in each subtype of MPM (12 epithelioid, 1 sarcomatoid, 10 biphasic, 2 other histological subtypes and 7 cytology only) were 63.0 (58.2), 8.2, 18.9 (24.8), 56.0 (61.0) and 104.4 (197.3), while the mean (standard deviation) CYFRA 21-1/CEA ratio in each subtype of MPM were 584.3 (11539), 731.8, 1379.6 (44140), 5204.7 (5170), 211.3(123.6). SMRP levels and the CYFRA 21-1/CEA ratio relative to PE diagnoses are shown in Table 3. We have also divided all the included patients into 4 disease subgroups (MPM, Lung cancer, Other malignancy and Others) and assessed SMRP levels and CYFRA 21-1/CEA ratio in each subgroup of patients using the complete-case dataset (Fig 2, Fig 3). In the complete-case dataset, the mean (standard deviation) SMRP level of the MPM patients was 63.3 (103.0) nmol/L, which was significantly greater than that of Lung cancer (12.0 (24.4) nmol/L) (p = 0.01), Other malignancy (14.1 (23.4) nmol/L) (p = 0.02) and Others (5.8 (4.1) nmol/L) (p = 0.007) (Fig 2). The mean (standard deviation) CYFRA 21-1/CEA ratio of the MPM patients was 1078 (2650), which was significantly greater than that of Lung cancer 47 (206) (p = 0.04) and Others 51 (267) in the complete-case dataset (p = 0.04) (Fig 3). The mean (standard deviation) CYFRA 21-1/CEA ratio of Other malignancy was 275 (1052), which had no statistically significant difference from that of the MPM patients (p = 0.15) (Fig 3).

**Table 2:** Pleural effusion SMRP levels and CYFRA 21-1/CEA ratio.

	MPM (n = 32)		Non-MPM (n = 208)	
	imputed set	complete-case set	imputed set	complete-case set
Age (years)		73.6 (9.9)		73.0 (11.8)
SMRP (nmol/l)	56.1 (97.8) ^a^	63.3 (103)	8.7 (25.2) ^b^	8.1 (14.4)
CYFRA21-1/CEA	1044 (2572) ^c^	1078 (2650)	63.1 (394) ^d^	67.9 (383)

Numbers indicate means (standard deviation).

Missing values were imputed (a = 4, b = 48, c = 1, d = 9).

MPM, malignant pleural mesothelioma; SMRP, soluble mesothelin-related peptides; CYFRA21-1/CEA, cytokeratin-19 fragments/carcinoembryonic antigen.

**Table 3:** Diagnosis, pleural effusion SMRP levels and CYFRA 21-1/CEA ratio.

	Patients, n	Age, yr	SMRP (nmol/L)	CYFRA 21-1/CEA ratio
MPM	32	74 (10)	28.0 [13.8–90.9] (n = 4)^a^	163.2 [37.1–731.8](n = 1)^a^
Lung cancer	44	71 (8)	5.5 [3.0–12.2] (n = 5)^a^	1.4 [0.2–5.7](n = 0)^a^
Other malignancy	17	64 (13)	5.4 [3.7–12.6] (n = 3)^a^	4.1 [0.7–20.4](n = 1)^a^
Unconfirmed malignant pleural effusion	2	79 (1)	6.3 [3.2–9.4] (n = 0)^a^	11.1 [1.4–20.7](n = 0)^a^
Benign asbestos related effusion	6	74 (6)	7.4 [3.6–9.1] (n = 1)^a^	16.5 [7.8–64.5](n = 0)^a^
Cardiac cause	14	83 (9)	5.1 [3.2–11.5] (n = 2)^a^	1.7 [0.9–9.5](n = 1)^a^
Non-cardiac transudate	14	75 (12)	5.9 [5.1–7.4] (n = 4)^a^	1.8 [0.8–4.6](n = 0)^a^
TB pleuritis	15	73 (15)	3.7 [2.3–5.8] (n = 3)^a^	13.9 [6.7–39.9](n = 0)^a^
Simple parapneumonic effusion	2	76 (5)	7.1 [–](n = 1)^a^	3.2 [2.0–4.3] (n = 0)^a^
Pleural infection	39	71 (13)	1.9 [1.2–6.1] (n = 14)^a^	12.0 [2.3–30.9](n = 6)^a^
Idiopathic pleuritis	8	68 (13)	4.4 [3.0–7.1] (n = 2)^a^	4.8 [2.0–23.6](n = 0)^a^
Undiagnosed	47	76 (10)	6.0 [4.3–9.0](n = 13)^a^	8.5 [3.7–25.4](n = 1)^a^
Total	240	73 (12)	5.7 [3.3–12.0](n = 52)^a^	7.2 [1.6–34.8](n = 10)^a^

Numbers indicate means (standard deviation).

Numbers indicate median [interquartile range].

^a^ Numbers indicate the number of patients with missing data.

MPM, malignant pleural mesothelioma; TB, tuberculosis.

### Chest CT findings for the diagnosis of MPM

The chest CT findings of MPM and non-MPM patients are presented in Table 4 (six of the non-MPM patients had no chest CT findings). Agreement between the two pulmonologists with regards to four features of pleural thickening (circumferential pleural thickening, nodularity, thickness>1cm, mediastinal pleural involvement) was assessed, and each of the kappa value was 0.93 (95%CI 0.90 to 0.97), 0.79 (95%CI 0.74 to 0.85), 0.84 (95%CI 0.79 to 0.88) and 0.71 (95%CI 0.65 to 0.77), respectively. The percentage of patients with these chest CT findings (circumferential pleural thickening, nodularity, thickness>1cm, mediastinal pleural involvement) was significantly higher in MPM than in non-MPM (22% and 7% (p<0.005), 69% and 20% (p<0.001), 50% and 14% (p<0.001), 66% and 30% (p<0.001)) (Table 4). The diagnostic utility of each chest CT finding is summarized in Table 5.

**Table 4:** Chest CT findings of included patients.

	MPM	Non-MPM	
	complete-case set (n = 32)	imputed set (n = 208)	complete-case set (n = 202)
Presence of Leung’s criteria	24 (75%)	75 (36%)	73 (36%)
Circumferential pleural thickening	7 (22%)	14 (7%)	13 (6%)
Nodularity	22 (69%)	41 (20%)	40 (20%)
Thickness >1cm	16 (50%)	30 (14%)	29 (14%)
Mediastinal pleural involvement	21 (66%)	63 (30%)	61 (30%)

Data are presented as n.

MPM, malignant pleural mesothelioma.

**Table 5:** The utility of chest CT findings and pleural effusion biomarkers in diagnosing malignant pleural mesothelioma.

	Sensitivity	Specificity	PPV	NPV
Chest CT findings				
Leung’s criteria	75.0 (56.6 to 88.5)	63.9 (57.0 to 70.5)	24.2 (16.2 to 33.9)	94.3 (89.1 to 97.5)
circumferential pleural thickening	21.9 (9.3 to 40.0)	93.3 (89.0 to 96.3)	33.3 (14.6 to 57.0)	88.6 (83.6 to 92.5)
Nodularity	68.8 (50.0–83.9)	80.3 (74.2–85.5)	34.9 (23.3 to 48)	94.4 (89.9 to 97.3)
thickness>1cm	50.0 (31.9–68.1)	85.6 (80.1–90.1)	34.8 (21.4 to 50.2)	91.8 (87.0 to 95.2)
mediastinal pleural involvement	67.7 (48.6–83.3)	69.7 (63.0–75.9)	25.0 (16.2 to 35.6)	93.5 (88.5 to 96.9)
Pleural effusion biomarkers				
SMRP	56.3 (50.0 to 73.6)	86.5 (81.1 to 90.9)	39.1 (25.1 to 54.6)	92.8 (88.2 to 96.0)
CYFRA 21-1/CEA ratio	87.5 (71.0 to 96.5)	74.0 (67.5 to 79.9)	34.1 (24.0 to 45.4)	97.5 (93.6 to 99.3)
SMRP or CYFRA21-1/CEA ratio	93.8 (79.2 to 99.2)	64.9 (58.0 to 71.4)	29.1 (20.6 to 38.9)	98.5 (94.8 to 99.8)
SMRP combined with chest CT findings				
SMRP or Leung’s criteria	84.4 (67.2 to 94.7)	51.4 (44.4 to 58.4)	21.1 (14.4 to 29.2)	95.5 (89.9 to 98.5)
SMRP or circumferential pleural thickening	68.8 (50.0 to 83.9)	82.7 (76.9 to 87.6)	37.9 (25.5 to 51.6)	94.5 (90.1 to 97.3)
SMRP or nodularity	81.3 (63.6 to 92.8)	69.2 (62.5 to 75.4)	28.9 (19.8 to 39.4)	96.0 (91.5 to 98.5)
SMRP or thickness>1cm	75.0 (56.6 to 88.5)	74.5 (68.0 to 80.3)	31.2 (21.1 to 42.7)	95.1 (90.6 to 97.9)
SMRP or mediastinal pleural involvement	81.3 (63.9 to 92.8)	60.6 (53.6 to 67.3)	24.1 (16.4 to 33.3)	95.5 (90.4 to 98.3)
CYFRA 21-1/CEA ratio combined with chest CT findings				
CYFRA 21-1/CEA ratio or Leung’s criteria	90.6 (75.0 to 98.0)	44.2 (37.4 to 51.3)	20.0 (13.8 to 27.4)	96.8 (91.0 to 99.3)
CYFRA 21-1/CEA ratio or circumferential pleural thickening	87.5 (71.0 to 96.5)	69.3 (62.7 to 75.5)	30.1 (21.0 to 40.5)	97.4 (93.4 to 99.3)
CYFRA 21-1/CEA ratio or nodularity	87.5 (71.0 to 96.5)	61.9 (55.1 to 68.4)	25.2 (17.5 to 34.4)	97.1 (92.8 to 99.2)
CYFRA 21-1/CEA ratio or thickness>1cm	87.5 (71.0 to 96.5)	63.0 (56.0 to 69.6)	26.7 (18.5 to 36.2)	97.0 (92.6 to 99.2)
CYFRA 21-1/CEA ratio or mediastinal pleural involvement	90.6 (75.0 to 98.0)	51.9 (44.9 to 58.9)	22.5 (15.6 to 30.7)	97.3 (92.3 to 99.4)
Pleural effusion biomarkers combined with chest CT findings				
SMRP or CYFRA 21-1/CEA ratio or Leung’s criteria	93.8 (79.2 to 99.2)	38.0 (31.4 to 45.0)	18.9 (13.1 to 25.8)	97.5 (91.4 to 99.7)
SMRP or CYFRA 21-1/CEA ratio or circumferential pleural thickening	93.8 (79.2 to 99.2)	61.1 (54.1 to 67.7)	27.0 (19.0 to 36.3)	98.4 (94.5 to 99.8)
SMRP or CYFRA 21-1/CEA ratio or nodularity	93.8 (79.2 to 99.2)	51.9 (44.9 to 58.9)	23.1 (16.1 to 31.3)	98.2 (93.6 to 99.8)
SMRP or CYFRA 21-1/CEA ratio or thickness>1cm	93.8 (79.2 to 99.2)	55.3 (48.3 to 62.2)	24.4 (17.1 to 33.0)	98.3 (94.0 to 99.8)
SMRP or CYFRA 21-1/CEA ratio or mediastinal pleural involvement	93.8 (79.2 to 99.2)	44.2 (37.4 to 51.3)	20.5 (14.3 to 28.0)	97.9 (92.5 to 99.7)

The analyzed data was based on imputed set (n = 240).

Data are presented as % (95% confidence intervals).

NPV, negative predictive value; PPV, positive predictive value; SMRP, soluble mesothelin-related peptides; CYFRA21-1/CEA, cytokeratin-19 fragments/carcinoembryonic antigen.

### SMRP and the CYFRA 21-1/CEA ratio for the diagnosis of MPM

The SMRP level in the PE of MPM and non-MPM patients was examined using a cut-off of 20 nmol/L, giving a sensitivity of 56.3% (95% CI 50.0 to 73.6%), specificity of 86.5% (95% CI 81.1 to 90.9%) for the diagnosis of MPM (Tables 5 and 6). The CYFRA 21-1/CEA ratio was examined at a cut-off value of 19.1, giving a sensitivity of 87.5% (95% CI 71.0 to 96.5%), a specificity of 74.0% (95% CI 67.5 to 79.9%) (Tables 5 and 7). The sensitivity and specificity of SMRP combined with the CYFRA 21-1/CEA ratio for the diagnosis of MPM were 93.8% (95% CI 79.2 to 99.2%) and 64.9% (95% CI 58.0 to 71.4%), respectively (Tables 5 and 8). In ROC curve analysis, the AUC for SMRP in analysis of its ability to distinguish MPM from all other PE causes was 0.804 (95% CI 0.690 to 0.918) while that of the CYFRA 21-1/CEA ratio was 0.874 (95% CI 0.780 to 0.948). There was no significant difference between the AUC for SMRP and the AUC for CYFRA 21-1/CEA (p = 0.13).

**Table 6:** SMRP levels in the pleural effusion of MPM and non-MPM patients.

SMRP	MPM	Non-MPM	Total
≥20.0 nmol/L	18 (17)	28 (7)	46 (24)
<20.0 nmol/L	14 (11)	180 (153)	194 (164)
Total	32 (28)	208 (160)	240 (188)

Data are presented as n (complete-case set).

SMRP, soluble mesothelin-related peptides; MPM, malignant pleural mesothelioma.

**Table 7:** The CYFRA21-1/CEA ratio of the pleural effusion of MPM and non-MPM patients.

CYFRA21-1/CEA ratio	MPM	Non-MPM	Total
≥19.1	28 (28)	54 (53)	82 (81)
**<**19.1	4 (3)	154 (146)	158 (149)
Total	32 (31)	208 (199)	240 (230)

Data are presented as n (complete-case set).

CYFRA21-1/CEA, cytokeratin-19 fragments/carcinoembryonic antigen; MPM, malignant pleural mesothelioma.

**Table 8:** Pleural effusion SMRP and the CYFRA21-1/CEA ratio in MPM and non-MPM patients.

SMRP (cut off ≥20.0 nmol/L) or CYFRA21-1/CEA ratio (cut off ≥19.1)	MPM	Non-MPM	Total
Positive	30 (27)	73 (47)	103 (74)
Negative	2 (1)	135 (113)	137 (114)
Total	32 (28)	208 (160)	240 (188)

Data are presented as n (complete-case set).

SMRP, soluble mesothelin-related peptides; CYFRA21-1/CEA, cytokeratin-19 fragments/carcinoembryonic antigen; MPM, malignant pleural mesothelioma.

### The sensitivity of chest CT findings combined with PE biomarkers

The diagnostic utility of chest CT findings combined with PE biomarkers is summarized in Table 5. When adding each chest CT finding (Leung’s criteria, circumferential pleural thickening, nodularity, thickness>1cm, mediastinal pleural involvement) to SMRP combined with the CYFRA 21-1/CEA ratio, the sensitivity was 93.8% (95% CI 79.2 to 99.2), 93.8% (95% CI 79.2 to 99.2), 93.8% (95% CI 79.2 to 99.2), 93.8% (95% CI 79.2 to 99.2), and 93.8% (95% CI 79.2 to 99.2) respectively, which indicated that the addition of chest CT findings did not increase the sensitivity of SMRP combined with the CYFRA 21-1/CEA ratio.

### SMRP and the CYFRA 21-1/CEA ratio in differentiating MPM and the other malignant tumors

As a subgroup analysis, we examined the diagnostic utility of SMRP and the CYFRA 21-1/CEA ratio in differentiating MPM and the other malignant tumors (lung cancer and other malignancy). SMRP and the CYFRA 21-1/CEA ratio had a sensitivity and specificity of 56.3% (95% CI 37.7 to 73.6) and 82.0% (95% CI 70.0 to 90.6), and 87.5% (95% CI 71.0 to 96.5) and 82.0% (95% CI 70.0 to 90.6), respectively. The sensitivity and specificity of SMRP combined with the CYFRA 21-1/CEA ratio were 93.8% (95% CI 79.2 to 99.2) and 72.1% (95% CI 59.2 to 82.9), respectively. In ROC curve analysis, the AUC for SMRP in analysis of its ability to distinguish MPM from the other malignant tumors was 0.784 (95% CI 0.679 to 0.889) while that of the CYFRA 21-1/CEA ratio was 0.883 (95% CI 0.805 to 0.961).

### Sensitivity analysis

**Fig 4:**
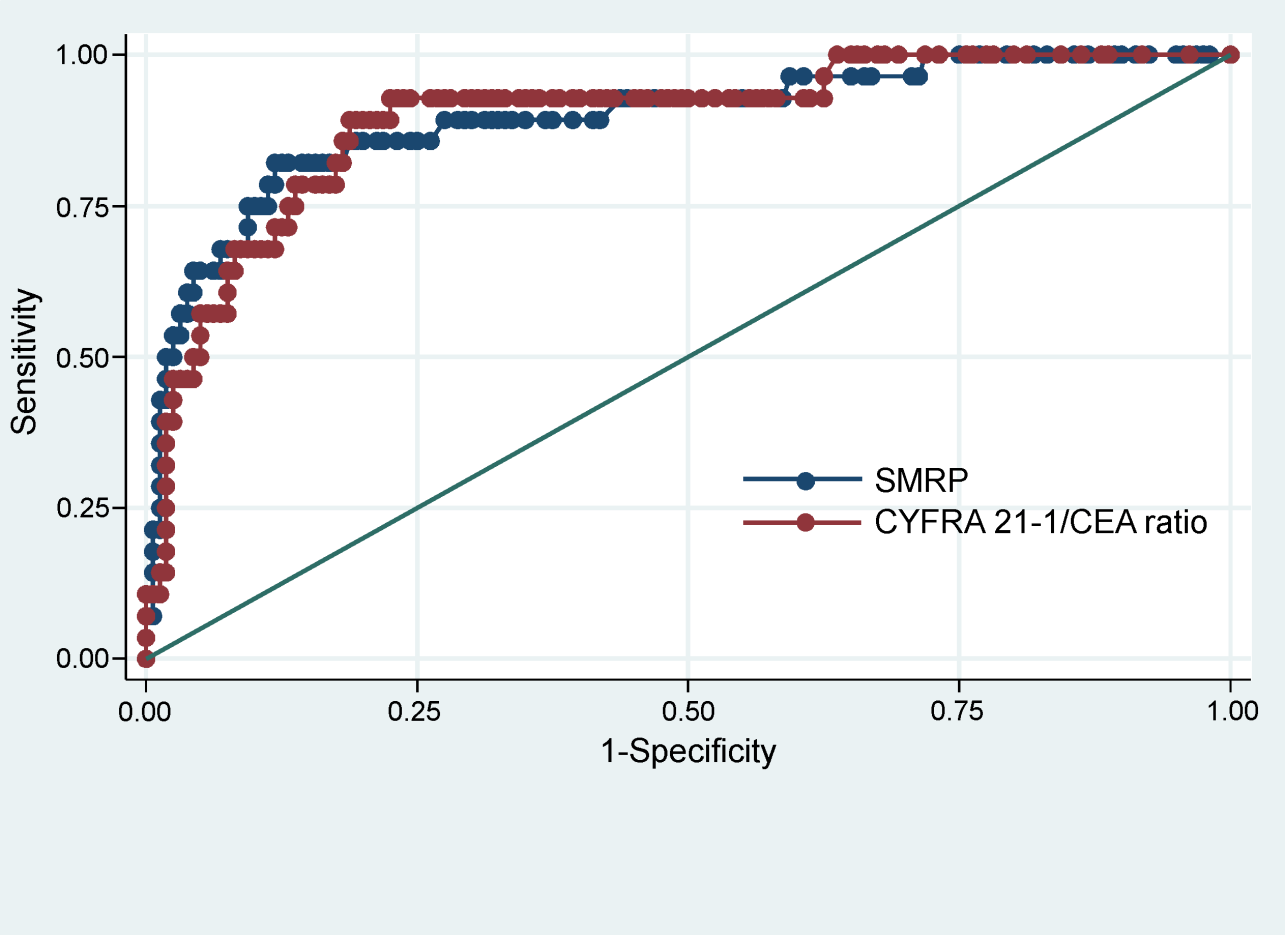
Receiver operating characteristic (ROC) curves of SMRP and CYFRA 21-1/CEA ratio.

We conducted sensitivity analysis using the complete-case set. In ROC curve analysis, the AUC for SMRP in analysis of its ability to distinguish MPM from all other PE causes was 0.895 (95%CI 0.824 to 0.965), while that of the CYFRA 21-1/CEA ratio was 0.894 (95%CI 0.829 to 0.959) (Fig 4). There was no significant difference between the AUC for SMRP and the AUC for CYFRA 21-1/CEA (p = 0.98). The sensitivity of SMRP, the CYFRA 21-1/CEA ratio, the combination of SMRP and the CYFRA 21-1/CEA ratio was 60.7% (95% CI 50.0 to 78.5%), 90.3% (95% CI 74.2 to 98.0%), 96.4% (95% CI 81.7 to 99.9%). When adding each chest CT finding (Leung’s criteria, circumferential pleural thickening, nodularity, thickness>1cm, mediastinal pleural involvement) to SMRP combined with the CYFRA 21-1/CEA ratio, the sensitivity was 96.4% (95% CI 81.7 to 99.9), 96.4% (95% CI 81.7 to 99.9), 96.4% (95% CI 81.7 to 99.9), 96.4% (95% CI 81.7 to 99.9), and 96.4% (95% CI 81.7 to 99.9) respectively.

## Discussion

This study showed that the combination of PE biomarkers (SMRP and the CYFRA 21-1/CEA ratio) had high sensitivity for diagnosing MPM, although the addition of chest CT findings did not increase the sensitivity of SMRP combined with the CYFRA 21-1/CEA ratio, both in the imputed set and in the complete-case set. Moreover, we found that the sensitivity of SMRP combined with the CYFRA 21-1/CEA ratio was also high in differentiating MPM and the other malignant tumors.

To the best of our knowledge, two previous reports have assessed the diagnostic accuracy of simultaneous SMRP, CYFRA 21–1 and CEA measurements when diagnosing MPM [9, 18]. According to one of those reports, SMRP differentiated MPM from non-MPM better than either CYFRA 21–1 or CEA (AUC = 0.84, 0.76, and 0.32 respectively; p = 0.003) [9]. However, our study showed that SMRP and the CYFRA 21-1/CEA ratio had almost the same ability to distinguish MPM from non-MPM (AUC = 0.804 and 0.874 respectively; p = 0.13), and that the combination of these two PE biomarkers had a relatively high sensitivity (93.8%) that could effectively rule out MPM.

Our study showed that the sensitivity of SMRP for the diagnosis of MPM was 56.3%, which was lower than the sensitivities of SMRP in the previous similar studies (around 70%) [1,4,11]. It is reported that pleural SMRP is overexpressed especially in the epithelioid MPM subtype [4], and pleural SMRP was significantly higher in epithelioid subtype compared with sarcomatoid subtype and biphasic subtype [11]. The relatively low sensitivity of SMRP in our study could be explained by the smaller percentage of epithelioid subtype (38%, 12 out of 32 MPM patients) compared to the previous reports above.

In other previous studies, hyaluronic acid was also reported as a possible pleural effusion biomarker for the diagnosis of MPM [28, 29]. In the recent report, hyaluronic acid had a sensitivity and specificity for diagnosing MPM of 44.0% and 96.5%, while it showed the AUC value of 0.832 for the differential diagnosis of MPM [28]. Therefore, it would be meaningful to assess the diagnostic utility of hyaluronic acid in combination with SMRP and CYFRA 21-1/CEA ratio in future studies.

Our study has four important clinical strengths compared to the two previous reports mentioned above [9,18]. First, this is the first study to examine the sensitivity of chest CT findings combined with these PE biomarkers. We found that chest CT findings did not increase the sensitivity of SMRP combined with the CYFRA 21-1/CEA ratio, although it did increase the sensitivity of the individual SMRP and CYFRA 21-1/CEA ratio biomarkers. Second, this study included consecutive inpatients and outpatients with undiagnosed PE, whereas the two previous studies included only patients admitted for PE diagnosis. In this respect, our study had a clinical strength because studies without inappropriate exclusions might prevent the overestimation of diagnostic accuracy [30]. Third, we used previously established cut-off levels of the PE biomarkers (SMRP, 20 nmol/L; CYFRA 21-1/CEA ratio, 19.1), whereas the two previous reports selected cut-off levels that were specific to their patient population. This means that our study prevented overoptimistic estimates of test performance [30]. Fourth, in this study, we assessed the CYFRA 21-1/CEA ratio, whereas the two previous reports above assessed CYFRA 21–1 and CEA individually. We assessed the CYFRA 21-1/CEA ratio because it has been reported that the use of this ratio improves the sensitivity in diagnosing MPM compared to the use of individual CYFRA 21–1 and CEA measurements [7].

The potential limitations of the present study are as follows. First, some of the patients who were analyzed with PE were excluded from this study (32 out of the 272 patients) (Fig 1). However, there was no significant difference in the percentage of MPM between excluded and included patients (22 and 13%), and it is therefore unlikely that these excluded patients affected the diagnostic ability of PE biomarkers in our study. Second, in this study, the reference standard (the diagnostic criteria of PE described in Table 1) could have introduced bias because the reference standard results were interpreted with knowledge of the results of the index test (chest CT findings) [30]. However, we think that this bias had little impact on the utility of PE biomarkers or chest CT findings in diagnosing MPM because the diagnosis of MPM in this study was based only on histological or cytological findings (Table 1). Third, pleural effusion SMRP levels and CYFRA 21-1/CEA ratios were missing in some of the patients in this study because of its retrospective design, and we used multiple imputation to handle missing data. However, we think that using multiple imputation in this study could be justified because it is reported that this method reduces bias from missing data and improves the precision of estimates [31]. Fourth, 7 of the MPM patients were diagnosed only by cytology, and their specific MPM subtypes could not be obtained. However, this did not influence the diagnostic ability of pleural effusion biomarkers in this study.

In summary, low levels of the PE biomarkers, SMRP and the CYFRA 21-1/CEA ratio, could strongly exclude the possibility of MPM. Assessment of the combination of these PE biomarkers is useful when ruling out MPM among patients at high risk of suffering MPM, and would be valuable especially for old frail patients who have difficulty in undergoing invasive procedures such as thoracoscopy.
